# The Ubiquitin Ligase RPM-1 and the p38 MAPK PMK-3 Regulate AMPA Receptor Trafficking

**DOI:** 10.1371/journal.pone.0004284

**Published:** 2009-01-27

**Authors:** Eun Chan Park, Doreen R. Glodowski, Christopher Rongo

**Affiliations:** The Waksman Institute, Department of Genetics, Rutgers University, Piscataway, New Jersey, United States of America; The Rockefeller University, United States of America

## Abstract

Ubiquitination occurs at synapses, yet its role remains unclear. Previous studies demonstrated that the RPM-1 ubiquitin ligase organizes presynaptic boutons at neuromuscular junctions in *C. elegans* motorneurons. Here we find that RPM-1 has a novel postsynaptic role in interneurons, where it regulates the trafficking of the AMPA-type glutamate receptor GLR-1 from synapses into endosomes. Mutations in *rpm-1* cause the aberrant accumulation of GLR-1 in neurites. Moreover, *rpm-1* mutations enhance the endosomal accumulation of GLR-1 observed in mutants for *lin-10*, a Mint2 ortholog that promotes GLR-1 recycling from Syntaxin-13 containing endosomes. As in motorneurons, RPM-1 negatively regulates the *pmk-3*/p38 MAPK pathway in interneurons by repressing the protein levels of the MAPKKK DLK-1. This regulation of PMK-3 signaling is critical for RPM-1 function with respect to GLR-1 trafficking, as *pmk-3* mutations suppress both *lin-10* and *rpm-1* mutations. Positive or negative changes in endocytosis mimic the effects of *rpm-1* or *pmk-3* mutations, respectively, on GLR-1 trafficking. Specifically, RAB-5(GDP), an inactive mutant of RAB-5 that reduces endocytosis, mimics the effect of *pmk-3* mutations when introduced into wild-type animals, and occludes the effect of *pmk-3* mutations when introduced into *pmk-3* mutants. By contrast, RAB-5(GTP), which increases endocytosis, suppresses the effect of *pmk-3* mutations, mimics the effect of *rpm-1* mutations, and occludes the effect of *rpm-1* mutations. Our findings indicate a novel specialized role for RPM-1 and PMK-3/p38 MAPK in regulating the endosomal trafficking of AMPARs at central synapses.

## Introduction

Synapses are the sites of cellular communication between presynaptic neurons and their postsynaptic partners. Presynaptic terminals contain multiple synaptic vesicles, which release neurotransmitter [1]. Receptors on the postsynaptic side of the synapse receive the neurotransmitter signals from the presynaptic cell. Changes in the localization and regulation of these receptors in turn mediate the changes in synaptic efficacy that occur during learning and memory [2]. The formation of presynaptic terminals and postsynaptic specializations is coordinated, but requires distinct sets of proteins.

Numerous regulators of presynaptic terminals at neuromuscular junctions (NMJs) have been identified. In particular, a conserved family of proteins, the PHR proteins (including vertebrate Phr1 and Pam, *Drosophila* Highwire, and *C. elegans* RPM-1), regulates the assembly of presynaptic components at NMJs [3]–[8]. PHR proteins contain multiple domains, including several RCC1 repeats, two repeats termed PHR domains, and a RING H2 domain. PHR proteins have both E3 ubiquitin ligase activity and guanine nucleotide exchange factor (GEF) activity, and can bind to Myc, adenylate cyclase, tuberin, the co-SMAD Medea, and dual leucine zipper kinases (DLKs) known to regulate the p38 MAP Kinase (MAPK) pathway [3], [9]–[14].

A particularly well-studied PHR protein is RPM-1, which regulates the formation of NMJs by two parallel mechanisms. First, RPM-1 forms an SCF-like complex with the F-box protein FSN-1, the SKP1 ortholog SKR-1, and the Cullin CUL-1; the resulting ubiquitin ligase ubiquitinates DLK-1, an upstream component of a *C. elegans* p38 MAPK pathway [14], [15]. Second, RPM-1 binds to GLO-4, an RCC1-like GEF that regulates GLO-1, a Rab GTPase [16]. RPM-1 is thought to positively regulate a Rab GTPase pathway to promote vesicular trafficking via late endosomes, which is critical for the organization of presynaptic terminals. Little is known about the function of PHR proteins like RPM-1 outside of the presynaptic terminal of NMJs, although they are abundantly expressed in the CNS.

The formation of postsynaptic specializations at excitatory central synapses has also been well studied. Ionotropic glutamate receptors (GluRs) form tetrameric channels on the postsynaptic face of central synapses, where they receive glutamatergic signals from the presynaptic cell [17]. The regulated trafficking of AMPA-type GluRs (AMPARs) into and out of the postsynaptic membrane is thought to underlie several forms of synaptic plasticity [18]–[21]. Ubiquitination and endocytosis are key mechanisms that regulate AMPAR postsynaptic accumulation [22]–[27]. However, the specific proteins that mediate the ubiquitin-dependent regulation of AMPARs are not well characterized.

To investigate these processes in a genetic system, we and others previously examined the trafficking of the GLR-1 AMPAR subunit in *C. elegans*. GLR-1 is expressed in the command interneurons, where it mediates nose-touch mechanosensation and regulates the frequency of spontaneous reversals in locomotion [28]–[30]. Functional GLR-1 receptors, fused with green fluorescent protein (GLR-1::GFP), are localized to punctate clusters at central synapses in the nerve ring (proximal neurites that encircle the pharynx) and along the ventral cord (the fascicle of distal neurites that run along the ventral midline) [31]. The synaptic abundance of GLR-1 is regulated by ubiquitination and endocytosis [24], [27], [32], [33]. GLR-1 synaptic abundance is also controlled by LIN-10, a PDZ-domain protein of the Mint family, which is thought to stimulate the membrane recycling of GLR-1 [31], [34]–[37]. Mutants that lack LIN-10 activity have decreased levels of punctate, synaptic GLR-1, and instead accumulate GLR-1 receptors in large, aberrant compartments at non-synaptic sites throughout their neurites [31], [37], [38]. Mutations that block endocytosis suppress the aberrant accumulation of GLR-1 in *lin-10* mutants, suggesting that GLR-1 is accumulating in an internal, post-endocytosis compartment within the neurites of these mutants [37]. Consistent with this model, *lin-10* mutants also have deficits in GLR-1-mediated behaviors, which can be suppressed by blocking endocytosis [37].

Here we report findings from our screen for genetic modifiers of *lin-10* mutations. We identify *rpm-1* as an enhancer of *lin-10*, as mutations in *rpm-1* enhance the aberrant accumulation of GLR-1 in neurites and the cell body that is observed in *lin-10* mutants. Mutants for *rpm-1* alone also accumulate GLR-1 in large compartments, although to a lesser extent than *lin-10* mutants. Whereas *rpm-1* mutants have disorganized presynaptic terminals at motorneuron NMJs, we find that presynaptic terminals at interneuron central synapses of *rpm-1* mutants are normal at a gross level. Restoration of *rpm-1* function in presynaptic neurons does not rescue the GLR-1 trafficking defects of *rpm-1* mutants. Instead, we find that RPM-1 functions in the postsynaptic interneurons that express GLR-1. As in the motorneurons, we find that RPM-1 regulates signaling via the PMK-3/p38 MAPK pathway in interneurons.

In addition, our findings point to the regulation of GLR-1 endocytosis as the mechanism for PMK-3 and RPM-1 function. Mutations that positively or negatively alter endocytosis both mimic and occlude the effects of *rpm-1* or *pmk-3* mutations, respectively, on GLR-1 trafficking to Syntaxin-13-containing endosomes. We propose that p38 MAPK stimulates GLR-1 endocytosis, and that RPM-1 inhibits p38 MAPK signaling, thereby acting to reduce GLR-1 endocytosis and to stabilize GLR-1 at the synapse. Our findings demonstrate a novel function for PHR proteins: the regulation of postsynaptic elements at central synapses via the regulation of endocytosis.

## Results

### RPM-1 Regulates GLR-1 Trafficking in Neurites

**Figure 1:**
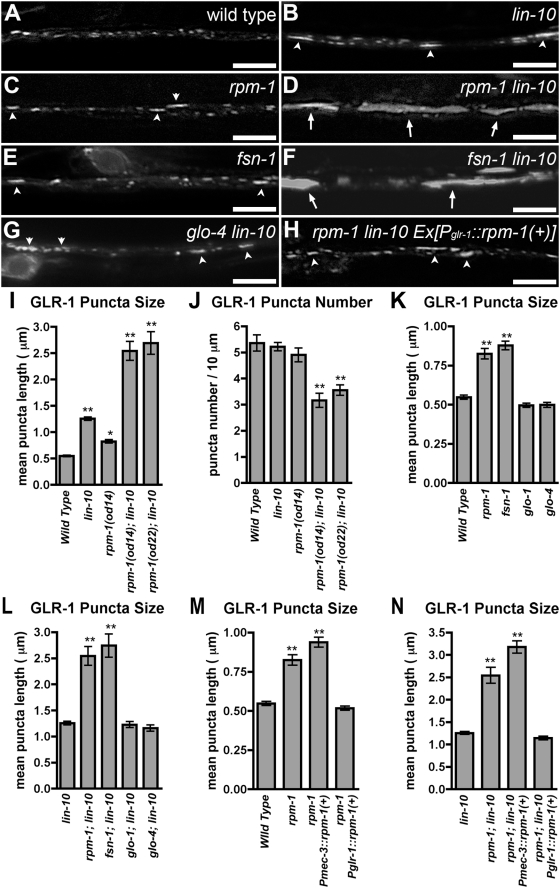
RPM-1 regulates GLR-1 trafficking. GLR-1::GFP fluorescence was observed along ventral cord neurites of (A) wild-type animals, (B) *lin-10* mutants, (C) *rpm-1* mutants, (D) *rpm-1 lin-10* double mutants, (E) *fsn-1* mutants, (F) *fsn-1 lin-10* double mutants, (G) *glo-4 lin-10* double mutants, or (H) *rpm-1 lin-10* double mutants that express a wild-type *rpm-1* minigene using the *glr-1* promoter. Arrowheads indicate the accumulation of GLR-1::GFP in large (1–5 micron long) accretions in *lin-10*, *rpm-1*, and *fsn-1* mutants. Arrows indicate the exaggerated accumulation of GLR-1::GFP in very large (>5 micron long) accretions in *rpm-1 lin-10* double mutants and *fsn-1 lin-10* double mutants. The mean size (I,K,L,M,N) and the mean number (J) of fluorescent structures (including puncta and accretions) are plotted for young adult nematodes of the given genotype. Bar, 5 µm. Error bars are SEM. N = 15–25 animals for each genotype. *P<0.01, **P<0.001 by ANOVA followed by Dunnett's Multiple Comparison to (I,J,K,M) wild type or to (L,N) *lin-10* mutants.

To better understand how GLR-1 is trafficked to synapses, we performed an EMS screen for suppressors and enhancers of a *lin-10* loss of function mutation as this mutation results in the aberrant accumulation of GLR-1 [37]. We identified two mutants, *od14* and *od22*, with an enhanced *lin-10* phenotype. Whereas wild-type animals contain punctate GLR-1::GFP at synapses along neurites (Fig. 1A), mutants for *lin-10* (Fig. 1B) or *rpm-1* (Fig. 1C) accumulate GLR-1 in large accretions found within the neurites. The shift in GLR-1 localization from small puncta to large accretions can be quantified by measuring mean puncta size in the ventral cord (Fig. 1I). Double mutants using molecular null alleles for *lin-10* and *rpm-1* have significantly larger accretions of GLR-1 than either single mutant alone (Fig. 1D,I), and have reduced numbers of small GLR-1 puncta compared to wild type (Fig. 1J), suggesting that these two genes might act in separate pathways to regulate GLR-1 trafficking.

**Figure 2:**
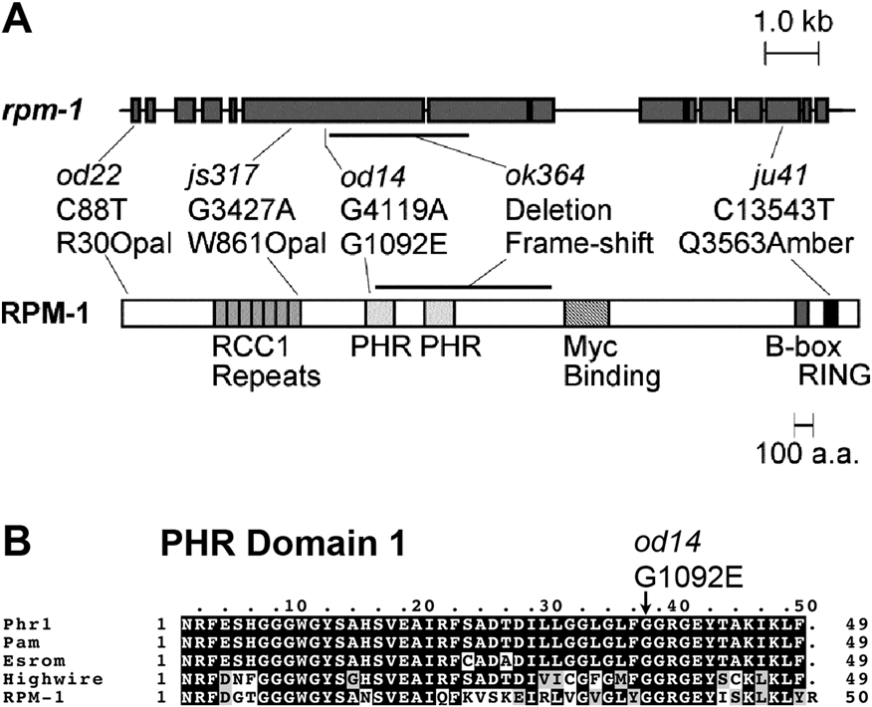
Mutations in RPM-1. (A) The intron/exon gene structure of *rpm-1* (gray boxes) is shown at top. At bottom is the predicted protein domain structure, including the RCC1 repeats, the dual PHR domains, the Myc-bind region, the B-box, and the RING domain. The molecular nature of several known alleles is indicated. (B) Amino acid alignment of mouse Phr1, human Pam, Zebrafish Esrom, Drosophila *highwire*, and *C. elegans* RPM-1. Black highlighting indicates common identities, and gray highlighting indicates similarities. In the *od14* mutation (indicated by the arrow), glycine is replaced with glutamate at a conserved PHR domain residue.

We identified *od14* and *od22* as alleles of *rpm-1* by genetic mapping and by their failure to complement known *rpm-1* alleles (Fig. 2A; see Supplementary Materials and Methods for details). We examined three other previously identified alleles of *rpm-1* (*ok364, ju41, js317*), and found that all three behaved like *od14* and *od22* with regard to GLR-1 localization (data not shown). To determine the molecular nature of our alleles, we sequenced genomic DNA from both mutants. The *od14* mutation alters glycine 1092 to a glutamate (Fig. 2A,B). This residue falls within the first PHR domain, and is conserved in all PHR protein family members, suggesting that glycine 1092 is critical for PHR domain function, and that the PHR domain is required to regulate GLR-1 trafficking. The *od22* mutation alters the conceptual translation of RPM-1 protein from arginine to an Opal stop codon at amino acid 30, resulting in a protein lacking all known functional domains (Fig. 2A).

As RPM-1 forms an E3 ubiquitin ligase complex with the F-box protein FSN-1 and the Cullin CUL-1 [15], we tested whether mutations in *fsn-1* result in a similar GLR-1 localization phenotype as that in *rpm-1*. The *fsn-1(hp1)* mutation introduces a stop codon before the SPRY domain, resulting in a protein null allele. Like *rpm-1* mutants, *fsn-1* mutants accumulate GLR-1 in large accretions (Fig. 1E,K). Moreover, mutations in *fsn-1* enhance the accumulation of GLR-1 observed in *lin-10* mutants (Fig. 1F,L) and depress the number of small GLR-1 puncta (data not shown).

RPM-1 is also known to act in motorneurons via GLO-4, an RCC1-like guanine nucleotide exchange factor that regulates GLO-1, a Rab GTPase [16]. We tested whether mutations in *glo-1* and *glo-4* result in a similar GLR-1 trafficking phenotype as that in *rpm-1*. Unlike mutations in *fsn-1*, mutations in *glo-1* and *glo-4* do not resemble *rpm-1* mutations; no GLR-1 accumulation was observed (Fig. 1K). In addition, mutations in either *glo-1* or *glo-4* do not enhance *lin-10* mutations (Fig. 1G,L). These results suggest that, unlike in motorneurons, RPM-1 does not regulate GLR-1 trafficking via GLO-1 and GLO-4. Instead, RPM-1 acts with FSN-1 to regulate GLR-1 trafficking, most likely as an E3 ligase.

### RPM-1 Has Different Roles in Different Neuron Types

**Figure 3:**
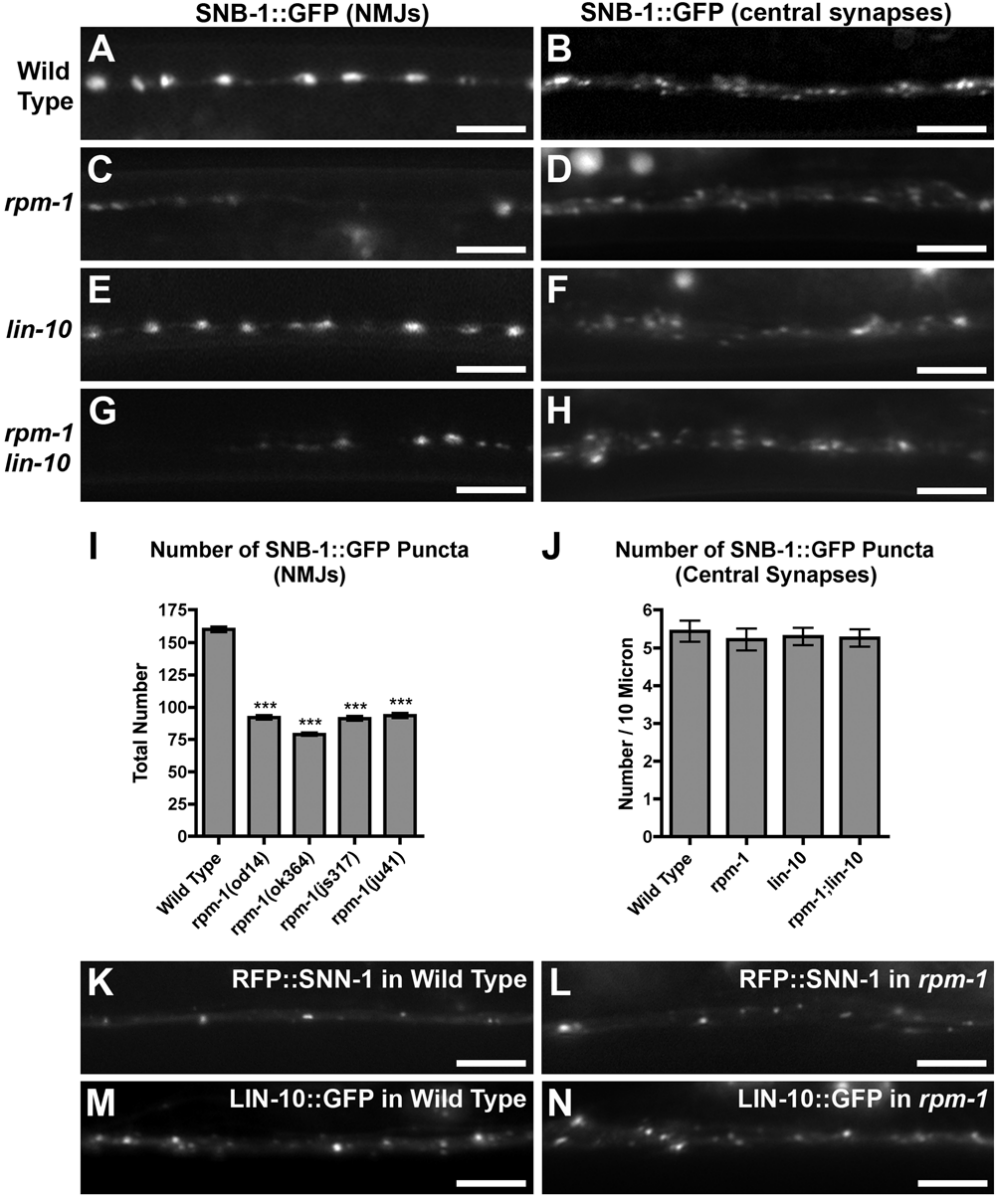
Differential requirements for RPM-1 at NMJs versus central synapses. SNB-1::GFP fluorescence was observed along the ventral cord of animals either (A,C,E,G) expressing the reporter at motorneuron NMJs via the *unc-25* promoter, or (B,D,F,H) expressing the reporter at interneuron central synapses via the *glr-1* promoter. SNB-1::GFP localization was observed in (A,B) wild-type animals, (C,D) *rpm-1* mutants, (E,F) *lin-10* mutants, or (G,H) *rpm-1 lin-10* double mutants. (I) The number of fluorescent structures (puncta) is plotted for motorneuron NMJs. (J) The mean number of puncta is plotted for interneuron central synapses. The fluorescence of (K,L) RFP::SNN-1 and (M,N) LIN-10::GFP, expressed from the *glr-1* promoter, were also observed along the ventral cord of (K,M) wild-type animals and (L,N) *rpm-1* mutants. Bar, 5 µm. Error bars are SEM. N = 15–25 animals for each genotype. ***P<0.001 by ANOVA followed by Dunnett's Multiple Comparison to wild type.

RPM-1 is required for proper presynaptic bouton formation at motorneuron NMJs [5], [6]. To determine whether our newly identified *rpm-1* mutations also impair presynaptic bouton formation, we examined the subcellular localization of SNB-1 (synaptobrevin) using *juIs1*, a transgene that expresses a SNB-1::GFP protein fusion in motorneurons. In wild-type animals, SNB-1::GFP is localized to large (1–2 micron) NMJ boutons along both the dorsal and ventral cords [14], [15], [39] (Fig. 3A). In *rpm-1(od14)* mutants, we observed a decreased number of SNB-1::GFP NMJ boutons and an irregularity in inter-bouton spacing, as was observed for other alleles of *rpm-1* mutants (Fig. 3C,I). Given the genetic interaction between *rpm-1* and *lin-10* with regard to GLR-1 localization, we examined SNB-1::GFP at the NMJ boutons of *lin-10* single mutants (Fig. 3E) and *rpm-1 lin-10* double mutants (Fig. 3G). NMJ boutons from *lin-10* mutants were indistinguishable from those in wild type, and *rpm-1 lin-10* double mutants were indistinguishable from those in *rpm-1* single mutants. We quantified the total number of the dorsal cord SNB-1::GFP boutons in *rpm-1(od14)* mutants, and found it to be similar to that observed in known alleles of *rpm-1* (Fig. 3I). As the *od14* mutation alters the first PHR domain, our findings suggest that the PHR domain is critical for RPM-1 function in motorneurons.

Given the changes in presynaptic boutons observed in *rpm-1* mutant motorneurons, we reasoned that the changes in GLR-1 ventral cord accumulation in *rpm-1* mutants could reflect general defects in synapse formation on interneurons. To test this possibility, we examined the localization of SNB-1 using *odIs1*, a transgene that expresses a SNB-1::GFP protein fusion in the GLR-1-expressing interneurons of the central nervous system. In wild-type animals, SNB-1::GFP is localized to small (∼0.5 micron) boutons along the ventral cord (Fig. 3B). In *rpm-1* mutants, there is no observable difference in SNB-1-labeled bouton number or size at central synapses (Fig. 3D). We also examined SNB-1-labeled central synapse boutons in *lin-10* single mutants (Fig. 3F) and *rpm-1 lin-10* double mutants (Fig. 3H), and found them to be indistinguishable from wild type (Fig. 3J). Finally, we examined the localization of a second presynaptic marker, RFP::SNN-1 (synapsin, [40]), at wild-type and *rpm-1* mutant interneuron synapses. RFP::SNN-1 is properly localized in both (Fig. 3K,L).

As *rpm-1* mutant interneurons lack the presynaptic defects observed in *rpm-1* mutant motorneurons, we reasoned that the postsynaptic GLR-1 trafficking defects in the *rpm-1* mutant neurons might instead be due to a cell-autonomous requirement for RPM-1 activity. We generated a transgene, *P_glr-1_::rpm-1(+)*, which contains *rpm-1* coding sequences under the control of the *glr-1* promoter. We introduced *P_glr-1_::rpm-1(+)* into *rpm-1* mutants, and found that it rescued (Fig. 1H,M). The ventral cord synapses that contain GLR-1 comprise a mixture of both interneuron-interneuron synapses and mechanosensory-interneuron synapses. Thus, we tested whether a transgene, *P_mec-3_::rpm-1(+),* which expresses RPM-1 in the mechanosensory neurons that innervate the GLR-1-expressing interneurons, could rescue *rpm-1* mutants [5]. *P_mec-3_::rpm-1(+)* rescues the sensory process branching defects of *rpm-1* mutant mechanosensory neurons ([5] and data not shown); however, it does not rescue the GLR-1 localization defects of the interneurons that are innervated by these same mechanosensory neurons (Fig. 1M).

As mutations in *rpm-1* behaved similarly to *lin-10* mutations with regard to their effect on GLR-1 trafficking, we also tested whether RPM-1 regulates LIN-10 localization. A LIN-10::GFP chimeric protein is colocalized with GLR-1 in the ventral cord in wild-type animals [31], [38]. We found that LIN-10::GFP is localized to similar punctate structures in both wild type and *rpm-1* mutants (Fig. 3M,N). Taken together, our findings indicate that the defects in GLR-1 localization observed in *rpm-1* mutants are unlikely to be due to gross defects in the formation of central synapses on the interneurons. In addition, our findings indicate that RPM-1 has a distinct role in organizing the presynaptic face of NMJ synapses in motorneurons but not central synapses in interneurons.

### PMK-3 and DLK-1 Act Downstream of RPM-1

**Figure 4:**
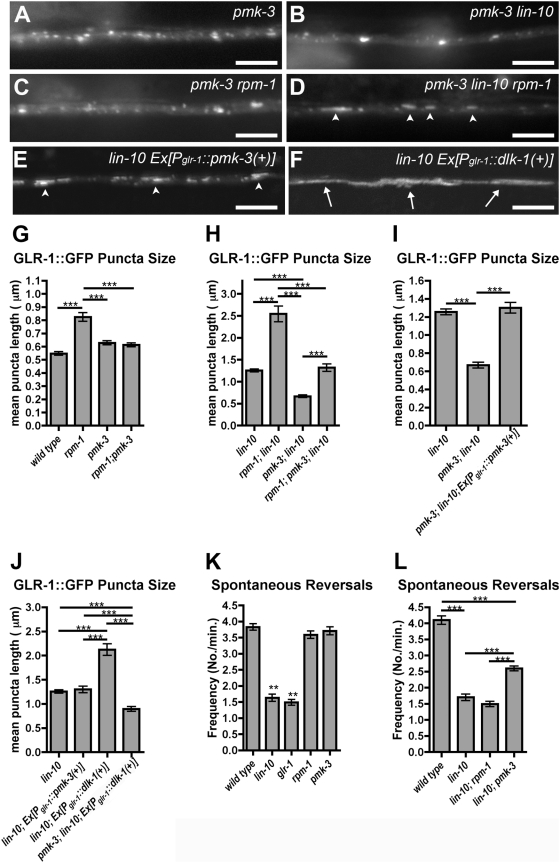
The PMK-3/p38 MAPK pathway modulates GLR-1 trafficking. GLR-1::GFP fluorescence was observed along ventral cord dendrites of (A) *pmk-3* mutants, (B) *pmk-3 lin-10* double mutants, (C) *pmk-3* *rpm-1* double mutants, (D) *pmk-3* *rpm-1 lin-10* triple mutants, (E) *lin-10* mutants that express a wild-type *pmk-3* cDNA using the *glr-1* promoter, or (F) *lin-10* mutants that express a wild-type *dlk-1* cDNA using the *glr-1* promoter. Arrowheads indicate the accumulation of GLR-1::GFP in large (1–5 micron long) accretions. Arrows indicate accumulation in very large (>5 micron long) accretions (similar to those found in *lin-10 rpm-1* double mutants). (G,H,I,J) The mean size of fluorescent structures (puncta and accretions) is plotted for adult nematodes of the given genotype. (K,L) The mean number of spontaneous reversals of locomotion for young adult animals is plotted for the given genotype. Bar, 5 µm. Error bars are SEM. N = 15–25 animals for each genotype. **P<0.01 by ANOVA followed by Dunnett's Multiple Comparison to wild type. ***P<0.001 by ANOVA followed by a Bonferroni Multiple Comparison test (indicated by lines).

One known target of RPM-1 regulation is the p38 MAPK cascade. To test for a role of p38 MAPK in GLR-1 trafficking, we examined *pmk-3(ok169)* mutants. The *pmk-3(ok169)* mutation is a deletion spanning most of the *pmk-3* coding sequence. Whereas the mutation in *pmk-3* did not affect the number or size of GLR-1 puncta in otherwise wild-type neurites (Fig. 4A), it did suppress the accumulation of GLR-1 observed in *lin-10* mutants, *rpm-1* mutants, and *rpm-1 lin-10* double mutants (Fig. 4B,C,D,G,H). These results indicate that PMK-3/p38 MAPK function is required for GLR-1 accumulation in *lin-10* and *rpm-1* mutants.

**Figure 5:**
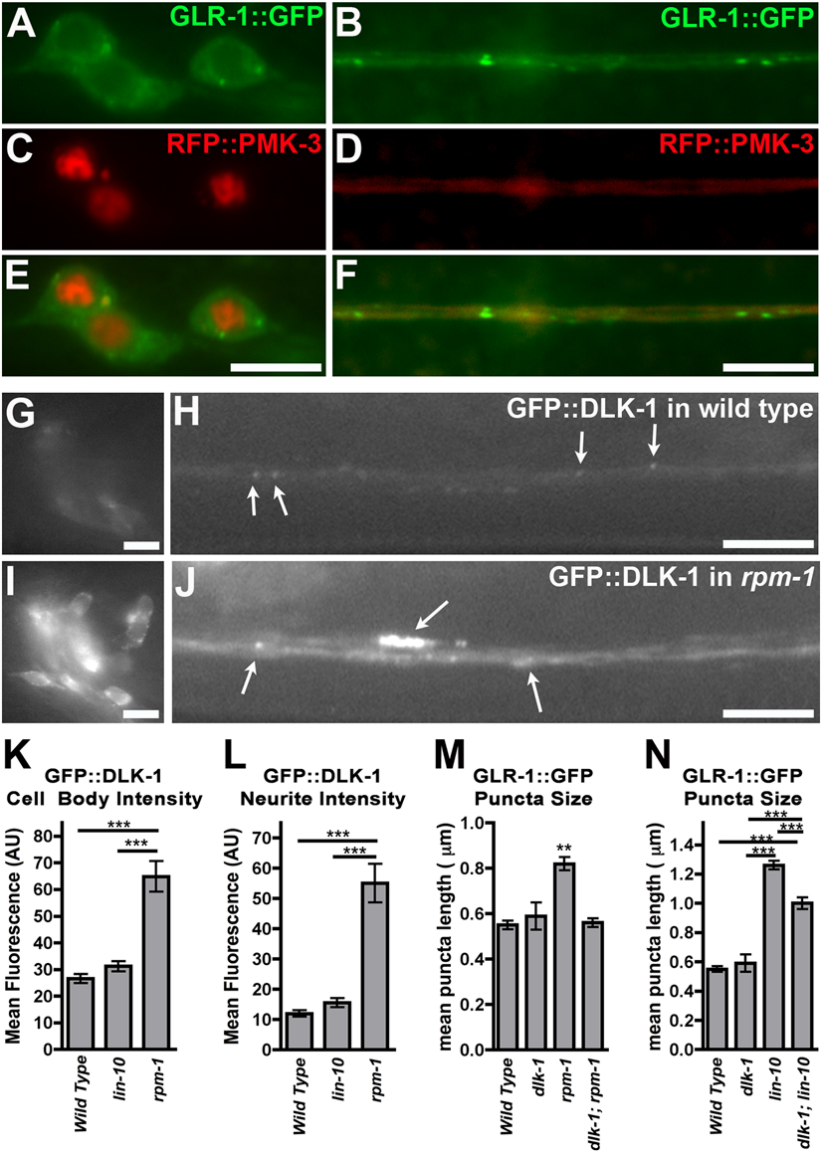
RPM-1 regulates DLK-1 levels in interneurons. (A,B) GLR-1::GFP or (C,D) RFP::PMK-3 fluorescence was observed in (A,C) the neuron cell bodies and (B,D) the ventral cord neurites of wild-type animals. (E,F) Merged images. GFP::DLK-1 fluorescence from the same transgenic line was observed in (G,I) the neuron cell bodies and (H,J) the ventral cord neurites of (G,H) wild-type animals and (I,J) *rpm-1* mutants. Arrows indicate puncta of GFP::DLK-1. (K,L) The mean GFP::DLK-1 fluorescence intensity for (K) neuron cell bodies and (L) ventral cord neurites is plotted for adult nematodes of the indicated genotype. (M,N) The mean size of fluorescent structures (puncta and accretions) is plotted for adult nematodes of the given genotype. Error bars are SEM. N = 20–25 animals for each genotype. *P<0.05, ***P<0.001 by ANOVA, followed by a Bonferroni Multiple Comparison test (indicated by lines).

PMK-3 is broadly expressed in many *C. elegans* tissues. To determine if PMK-3 functions in the same cells as GLR-1, we made a transgene, *P_glr-1_:: pmk-3(+)*, containing *pmk-3* cDNA sequences (with mRFP sequences fused in frame at the PMK-3 N-terminus) under the control of the *glr-1* promoter. We introduced *Pglr-1::pmk-3(+)* into *pmk-3 lin-10* double mutants and found that mutant animals carrying the transgenic array had their GLR-1 localization defects restored (Fig. 4I), indicating that RFP::PMK-3 can function cell autonomously. To examine PMK-3 subcellular localization, we introduced *P_glr-1_::pmk-3(+)* into wild-type animals expressing GLR-1::GFP, and found that RFP::PMK-3 protein was localized to interneuron nuclei (Fig. 5C) and was present throughout the ventral cord neurites (Fig. 5D).

Given the nuclear localization of PMK-3, we hypothesized that PMK-3 might regulate the transcription of *glr-1* mRNA. We isolated total mRNA from wild type, *pmk-3* mutants, *rpm-1* mutants, *lin-10* mutants, *pmk-3 lin-10* double mutants, *rpm-1 lin-10* double mutants, and *pmk-3 rpm-1 lin-10* triple mutants. We measured *glr-1* mRNA levels by real time PCR (using *snb-1* mRNA levels as a control), and detected no significant differences among these different genotypes (data not shown). Removing one of the two copies of the *nuIs25[glr-1::gfp]* transgene, which results in two-fold mRNA level differences that are detectable by real time PCR, does not suppress the GLR-1 accumulation observed in *lin-10* mutants [31]. Thus, it is unlikely that PMK-3 and RPM-1 regulate GLR-1 trafficking by affecting *glr-1* mRNA levels.

As RPM-1, an ubiquitin ligase, is a negative regulator of the p38 MAPK cascade, the abundance of one or more components of the cascade is likely to be limiting within neurons. To determine if the levels of PMK-3 are limiting, we introduced the *Pglr-1::pmk-3(+)* transgene into animals that also contained both wild-type alleles of the endogenous *pmk-3* locus. The elevation of PMK-3 levels in wild-type animals did not affect GLR-1 localization (data not shown). Also, *lin-10* mutants that contained elevated levels of PMK-3 did not demonstrate the dramatic enhancement observed in *rpm-1 lin-10* double mutants (Fig. 4E,J).

The PMK-3/p38 MAPK is regulated by the MAPK Kinase MKK-4, and mutations in *mkk-4*, like those in *pmk-3*, suppress the GLR-1 trafficking defects of *rpm-1* mutants (data not shown). MKK-4 in turn is activated by DLK-1, an upstream MAPKKK regulated directly by RPM-1 in motorneurons [14]. We generated a transgene, *P_glr-1_::dlk-1(+)*, containing *dlk-1* cDNA sequences (with mRFP sequences fused in frame at the DLK-1 N-terminus) under the control of the *glr-1* promoter. Unlike animals expressing *Pglr-1::pmk-3(+)*, animals expressing *P_glr-1_::dlk-1(+)* accumulated GLR-1 accretions in their neurites (data not shown). Moreover, *lin-10* mutants that contained elevated levels of DLK-1 demonstrated a dramatic enhancement in phenotype (Fig. 4F,J), similar to that observed in *rpm-1 lin-10* double mutants. The removal of PMK-3 activity from *lin-10 P_glr-1_::dlk-1(+)* animals blocked the effect of DLK-1 overexpression (Fig. 4J). Finally, a loss of function mutation in *dlk-1* suppressed the accumulation of GLR-1 observed in *rpm-1* and *lin-10* mutants (Fig. 5M,N). Our results indicate that PMK-3 levels are not limiting for GLR-1 localization; rather, the levels of the upstream MAPKKK DLK-1 appear to dictate PMK-3/p38 MAPK activity in controlling GLR-1 trafficking.

In *C. elegans*, GLR-1 signaling positively regulates spontaneous reversals during forward locomotion as animals forage for food [41], [42]. Mutants with either reduced GLR-1 activity (e.g., *glr-1* mutants) or reduced levels of GLR-1 receptor at the synaptic membrane surface (e.g., *lin-10* mutants) have a lower frequency of spontaneous reversals [37]. We examined GLR-1-mediated behaviors in wild-type animals and in animals with mutations in *lin-10, glr-1*, *rpm-1*, or *pmk-3*. We found that wild-type animals spontaneously reversed about 3.7 times per minute (20 animals, 5 minute trial per animal), whereas *lin-10* and *glr-1* mutants only spontaneously reversed direction about 1.5 and 1.4 times per minute, respectively (Fig. 4K). Mutants for either *rpm-1* or *pmk-3* spontaneously reversed direction with a frequency similar to that of wild-type animals. Interestingly, double mutants for *lin-10* and *pmk-3* reversed direction 2.5 times per minute (Fig. 4L), suggesting that mutations in *pmk-3* can partially restore GLR-1-mediated reversal behavior to *lin-10* mutants. Thus, the effect of *pmk-3* mutations on GLR-1 subcellular localization correlates with behaviors that reflect the synaptic strength of the interneuron reversal circuit.

### RPM-1 Regulates DLK-1 Abundance in Interneurons

If DLK-1 levels are limiting with regard to the regulation of GLR-1 trafficking, then RPM-1 might regulate DLK-1 levels. To observe changes in DLK-1 protein levels using a more sensitive reporter than our mRFP::DLK-1 chimera, we generated a transgene, *P_glr-1_::gfp::dlk-1(+)*, containing GFP sequences fused in frame to *dlk-1* cDNA sequences at the DLK-1 N-terminus, under the control of the *glr-1* promoter. We introduced the transgene into wild-type animals and found that GFP::DLK-1 protein was present both in neuron cell bodies (near the membrane and excluded from the nucleus; Fig. 5G) and in ventral cord neurites in a punctate pattern (Fig. 5H). We crossed the transgene into *rpm-1* mutants and observed a significant increase in GFP::DLK-1 fluorescence (the same transgenic line is shown in Fig. 5G,H,I,J). To quantify GFP::DLK-1 fluorescence, we removed background fluorescence from our images, then defined either whole cell bodies or whole ventral cords as single objects. We quantified the mean fluorescence for these objects and found a several fold increase in mean GFP::DLK-1 fluorescence in *rpm-1* mutants compared to wild type (Fig. 5K,L; same transgenic line is quantified). These differences were observed in both neuron cell bodies and ventral cord neurites. Mutations in *lin-10* had little effect on GFP::DLK-1 (Fig. 5K,L). Our findings suggest that RPM-1 negatively regulates DLK-1 protein levels in the command interneurons.

### RPM-1 Is Required for the Ubiquitin-Mediated Turnover of GLR-1

**Figure 6:**
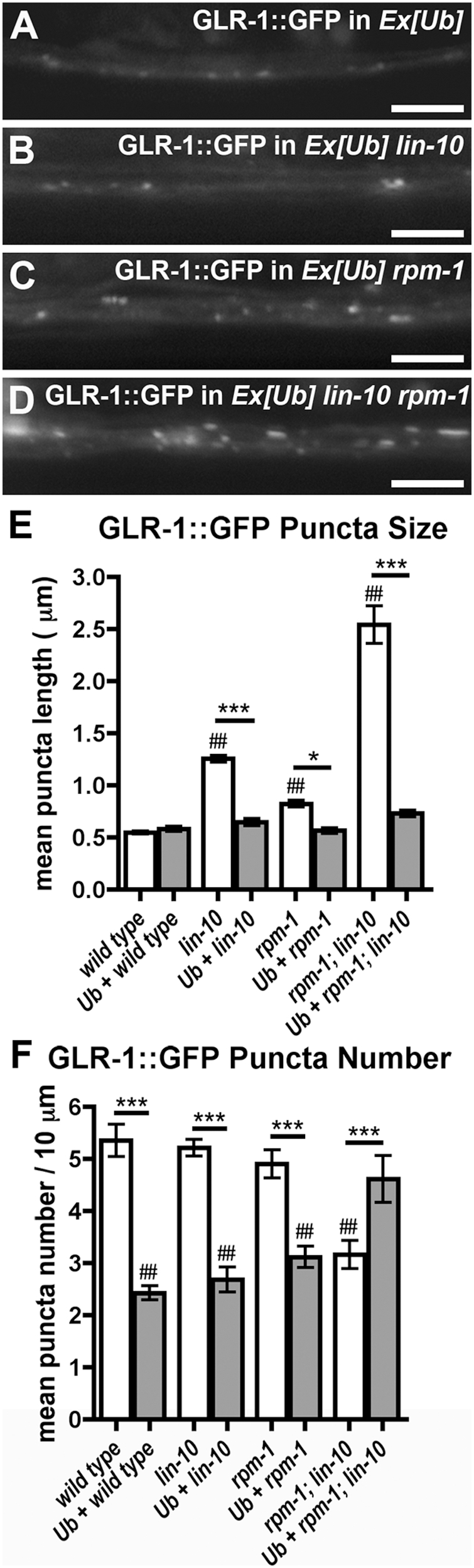
RPM-1 is required for the ubiquitin-mediated turnover of GLR-1. GLR-1::GFP fluorescence was observed along ventral cord dendrites of (A) wild-type animals, (B) *lin-10* mutants, (C) *rpm-1* mutants, or (D) *rpm-1 lin-10* double mutants, all of which also express ubiquitin from the *nuIs89* transgene. The mean size (E) and the mean number (F) of fluorescent structures (puncta) are plotted for adult nematodes of the given genotype. Bar, 5 µm. Error bars are SEM. N = 15–25 animals for each genotype. *P<0.05, ***P<0.001 by ANOVA followed by a Bonferroni Multiple Comparison test (indicated by line). ##P<0.001 by ANOVA followed by Dunnett's Multiple Comparison to wild type.

GLR-1 trafficking and synaptic abundance are regulated by ubiquitination. Because of a limiting cellular concentration of monoubiquitin, overexpression of Myc epitope-tagged ubiquitin (MUb) by a *nuIs89* transgene has been shown to negatively regulate GLR-1 abundance in neurites [24]. While multiple E3 ligases have been shown to be partially required for the turnover of GLR-1 after an overexpression of ubiquitin, mutations in no single E3 ligase have been shown to completely block GLR-1 turnover, suggesting that multiple ligases are involved. To determine if RPM-1 is required for the ubiquitin-mediated turnover of GLR-1, we introduced *nuIs89* into *lin-10* mutants, *rpm-1* mutants, and *rpm-1 lin-10* double mutants. Elevated ubiquitin depresses the number of GLR-1 puncta in wild-type animals (Fig. 6A,F). Mutations in *lin-10* and *rpm-1* combined block the effect of overexpressed ubiquitin on GLR-1 puncta number (Fig. 6D,F), indicating that these genes are required for some of the ubiquitin-mediated removal of GLR-1. However, in *lin-10* and *rpm-1* single mutants, as well as in *lin-10 rpm-1* double mutants, elevated ubiquitin can nevertheless still depress the size of GLR-1 accretions (Fig. 6B,C,D,E), suggesting that the GLR-1 receptors that accumulate internally in these mutants can still be removed in part by other ubiquitin-dependent mechanisms. Thus, RPM-1 is required for part, but not all, of the ubiquitin-mediated degradation of GLR-1.

### RPM-1 Regulates GLR-1 Endocytosis

**Figure 7:**
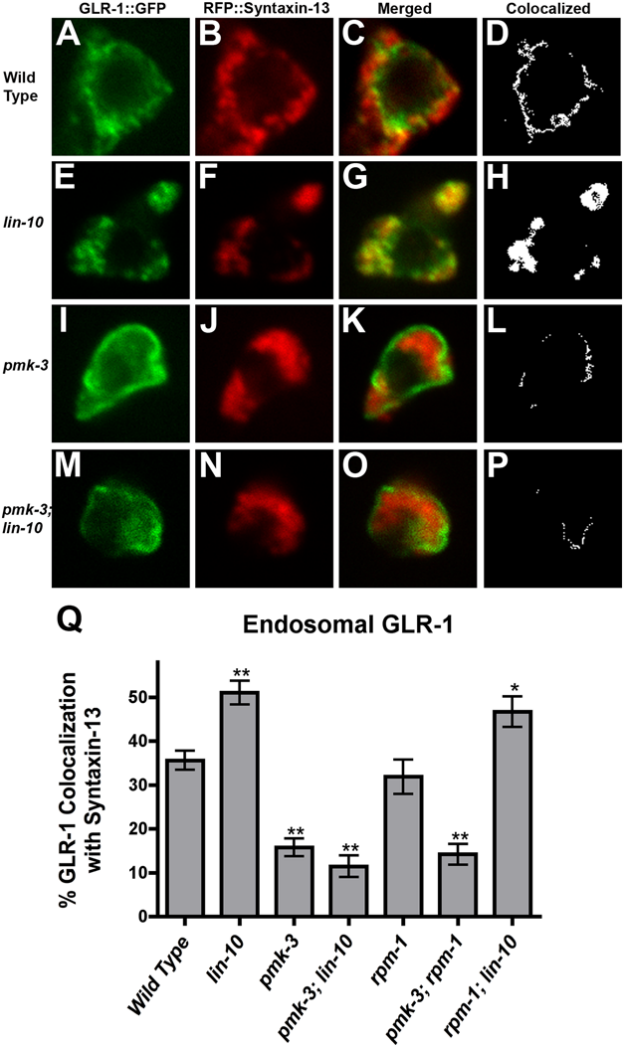
GLR-1 colocalization with endosome marker Synxtaxin-13. (A,E,I,M) GLR-1::GFP and (B,F,J,N) RFP::Syntaxin-13 fluorescence was observed in single confocal images of neuron cell bodies from (A–D) wild-type animals, (E–H) *lin-10* mutants, (I–L) *pmk-3* mutants, and (M–P) *pmk-3 lin-10* double mutants. (C,G,K,O) Merged images. (D,H,L,P) Binary masks (white) were created to highlight pixels with matching intensity values for both GLR-1::GFP and RFP::Syntaxin-13, indicating colocalization. (Q) The mean percent of GLR-1::GFP colocalized with Syntaxin-13 (endosomal), normalized to total cell body GLR-1::GFP. Error bars are SEM. *P<0.05, **P<0.01 by ANOVA followed by Dunnett's Multiple Comparison to wild type.

Previous studies have shown that p38 MAPK can modulate endocytosis in other systems, raising the possibility that PMK-3 regulates GLR-1 endocytosis [43]–[45]. Indeed, the accumulation of GLR-1 receptors into large accretions in *lin-10* mutants is due to defects in GLR-1 recycling from endosomes to synapses, suggesting that the large accretions are endosomes swollen with trapped receptors [37]. Direct colocalization with endosomal markers in the ventral cord has been difficult: fluorescently tagged endosomal residents have not been bright enough to visualize endosomes in the neurites ([37], [46] and our own unpublished observations). Recently, Chun et al demonstrated that an mRFP-tagged Syntaxin-13, a resident of early endosomes, colocalizes with GLR-1::GFP in neuron cell bodies [46]. In this study, *unc-108/rab-2* trafficking mutants, which contain mislocalized GLR-1 in large accretions in the ventral cord (similar to *lin-10*), also accumulated GLR-1 in Synxtaxin-13-labeled endosomes in the cell body. Using the *glr-1* promoter, we coexpressed RFP::Syntaxin-13 with GLR-1::GFP in wild-type animals, as well as in *lin-10* and *pmk-3* mutants, then observed subcellular localization using confocal microscopy. GLR-1::GFP is localized in small tubule-like structures inside neuron cell bodies, and a significant fraction of these structures colocalize with RFP::Syntaxin-13 (Fig. 7A–D,Q). In *lin-10* mutants, GLR-1::GFP accumulates in large accretions in the cell bodies, and these show more colocalization with RFP::Syntaxin-13 (Fig. 7E–H,Q). By contrast, *pmk-3* mutant neuron cell bodies accumulate GLR-1 at or near the outer membrane, with significantly less colocalization with RFP::Syntaxin-13 (Fig. 7I–L,Q). Moreover, mutations in *pmk-3* suppress the accumulation and colocalization of GLR-1 with Syntaxin-13 in *lin-10* mutants (Fig. 7M–P,Q). These results indicate that PMK-3 and LIN-10 have opposite effects on the accumulation of GLR-1 in endosomes.

**Figure 8:**
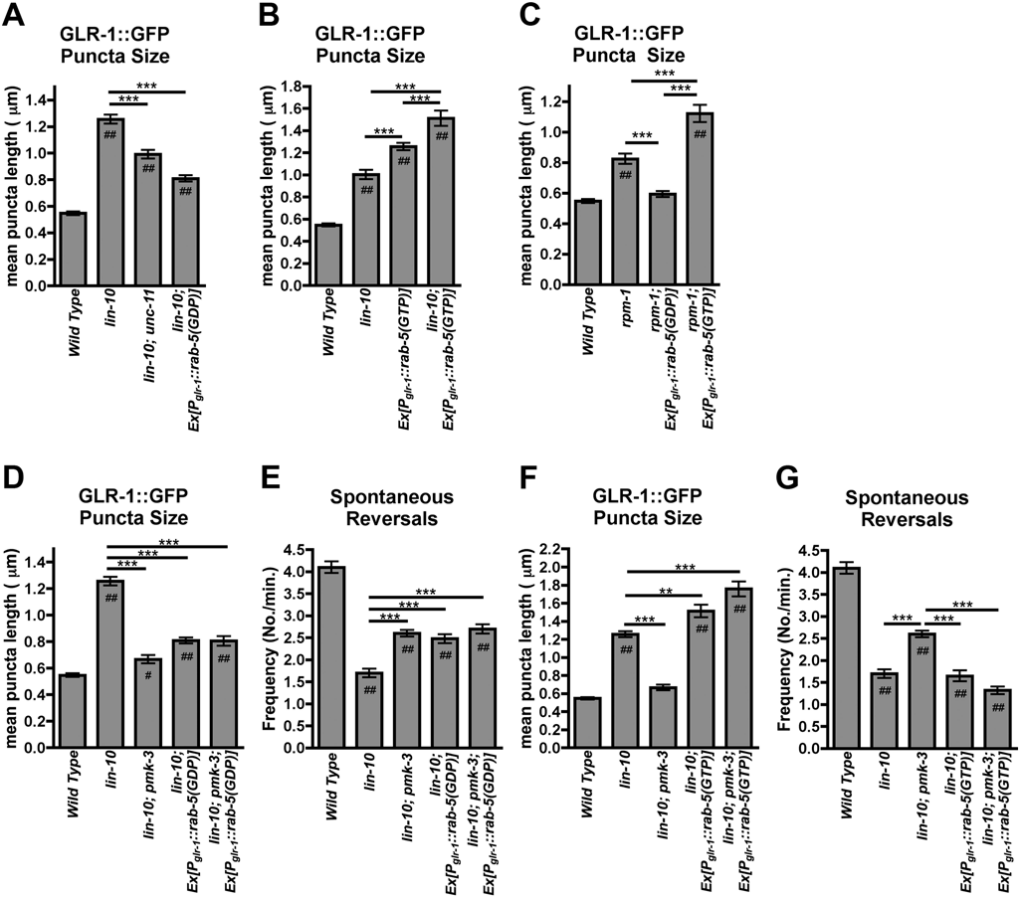
RPM-1 and PMK-3 regulate GLR-1 endocytosis. (A,B,C,D,F) The mean size of fluorescent structures (puncta and accretions) and (E,G) the mean reversal frequency is plotted for adult nematodes of the given genotype. (A) Decreased or (B) increased endocytosis suppresses or enhances the accumulation of GLR-1 in *lin-10* mutants, respectively, as well as (C) *rpm-1* mutants. Decreased endocytosis by (D,E) expression of *rab-5(GDP),* can mimic and occlude the effect of *pmk-3* mutations on GLR-1 trafficking. Increased endocytosis by (F,G) expression of *rab-5(GTP)* can block the effect of *pmk-3* mutations, thus bypassing the requirement for PMK-3. Error bars are SEM. N = 15–25 animals for each genotype. Solid lines with ** (P<0.01) or *** (P<0.001) indicate specific comparisons by ANOVA followed by a Bonferroni Multiple Comparison test. Comparisons to the wild-type control are indicated by # (P<0.05) or ## (P<0.01) using ANOVA followed by Dunnett's test.

The abnormal accumulation of GLR-1 in *lin-10* mutants can be suppressed by blocking clathrin-mediated endocytosis, further supporting that the receptors are trapped in endosomes in these mutants [37]. Mutations in *unc-11*, a clathrin adaptin protein AP180 ortholog, depress GLR-1 endocytosis [24]. When introduced into *lin-10* mutants, *unc-11* mutations suppressed the accumulation of GLR-1 in accretions (Fig. 8A). Endocytosis is also stimulated by the small GTPase RAB-5, which cycles between an active GTP-bound form and an inactive GDP-bound form [47]–[49]. We generated a transgene, *P_glr-1_::rfp::rab-5(GDP)*, which contains a mutated form of the *rab-5* cDNA expressed from the *glr-1* promoter. RAB-5(GDP) contains an S23N substitution, locking it in the GDP-bound conformation and depressing endocytosis. Like mutations in *unc-11*, expression of *P_glr-1_::rfp::rab-5(GDP)* in *lin-10* mutants suppressed the accumulation of GLR-1 in accretions (Fig. 8A). We also generated *P_glr-1_::rfp::rab-5(GTP)*, which contains *rab-5* cDNA with the mutation Q78L. RAB-5(GTP) is locked in the GTP-bound conformation and stimulates endocytosis. When we introduced *P_glr-1_::rfp::rab-5(GTP)* into wild-type animals, we found that GLR-1 accumulated in accretions, similar to those in *lin-10* mutants (Fig. 8B). Moreover, *P_glr-1_::rfp::rab-5(GTP),* when placed into *lin-10* mutants, significantly enhanced the accumulation of GLR-1 (Fig. 8B). Expression of *P_glr-1_::rfp::rab-5(GDP)* or *P_glr-1_::rfp::rab-5(GTP)* also suppressed or enhanced the accumulation of GLR-1 in accretions in *rpm-1* mutants, respectively (Fig. 8C). Thus, the net accumulation of GLR-1 in accretions in *lin-10* and *rpm-1* mutants depends on GLR-1 endocytosis.

To determine whether PMK-3/p38 MAPK regulates GLR-1 endocytosis, we tested whether suppression of *lin-10* by *pmk-3* mutations and by *unc-11* mutations was additive. We generated *lin-10 pmk-3 unc-11* triple mutants and examined GLR-1 accumulation. We found that *pmk-3* mutations did not enhance the phenotype of *unc-11* mutations (data not shown), suggesting that mutations in *unc-11* can occlude the effect of mutations in *pmk-3*. Similarly, we depressed endocytosis in *lin-10 pmk-3* double mutants by introducing the *P_glr-1_::rab-5(GDP)* transgene. We found that the expression of *P_glr-1_::rab-5(GDP)* occluded the effect of *pmk-3* mutations on GLR-1 trafficking (Fig. 8D). In addition, the expression of *P_glr-1_::rab-5(GDP),* was able to restore GLR-1-mediated spontaneous reversals to *lin-10* mutants to the same extent as the presence of a *pmk-3* mutation (Fig. 8E). Furthermore, the expression of *P_glr-1_::rab-5(GDP)* occluded the effect of a *pmk-3* mutation (Fig. 8E). These results are consistent with RAB-5 and PMK-3 functioning in the same pathway to stimulate GLR-1 endocytosis.

If PMK-3 regulates GLR-1 endocytosis by activating RAB-5, then expression of RAB-5(GTP) should bypass the requirement for PMK-3. To test this idea, we introduced *P_glr-1_::rab-5(GTP)* into *lin-10 pmk-3* double mutants and observed the effect on GLR-1 accumulation in accretions. We found that *P_glr-1_::rab-5(GTP)* enhanced the accumulation of GLR-1 in accretions, regardless of the presence of PMK-3 (Fig. 8F). Expression of *P_glr-1_::rab-5(GTP)* in *lin-10* mutants also depressed the spontaneous reversal frequency, even in combination with a *pmk-3* mutation (Fig. 8G). Taken together, our results suggest that PMK-3/p38 signaling regulates GLR-1 receptors by stimulating their endocytosis.

## Discussion

**Figure 9:**
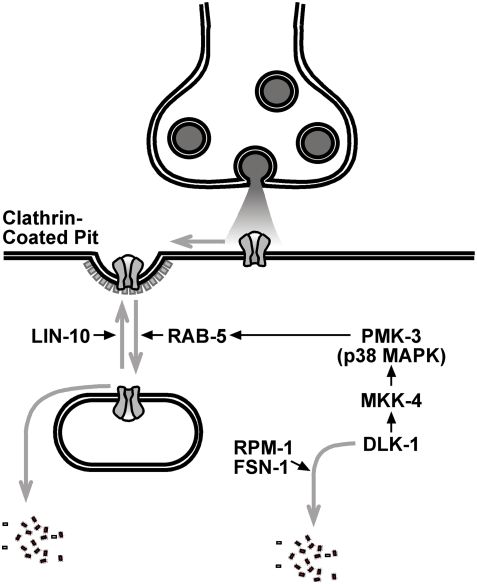
A model for the regulation of GLR-1 AMPAR trafficking by PMK-3 and RPM-1. GLR-1 (gray channels) endocytosis can occur via clathrin (pit indicated to left of the synapse). Gray arrows indicate major trafficking steps, positively regulated by the factor(s) indicated next to the arrows. Black arrows indicate positive (stimulatory) genetic regulatory interactions between two factors. GLR-1 endocytosis is mediated by UNC-11/AP180 and RAB-5. Once endocytosed, receptors can either be recycled to the synapse in a step requiring LIN-10, or degraded. PMK-3/p38 MAPK stimulates GLR-1 endocytosis via RAB-5 activation. PMK-3 is activated by a MAP kinase cascade, which includes MKK-4 and DLK-1. RPM-1 and FSN-1, working as an E3 ligase, negatively regulate DLK-1 (and hence p38 MAPK signaling) by ubiquitin-mediated turnover.

We identified a novel role for RPM-1 and PMK-3 as regulators of AMPAR endocytosis. Previously, LIN-10 was shown to mediate the recycling of GLR-1 from endosomes back to the synapse [37]. Mutations in *lin-10* result in the accumulation of GLR-1 in endosomes; this accumulation is suppressed by mutations that decrease endocytosis and enhanced by mutations that increase endocytosis. Several lines of evidence suggest that RPM-1 and PMK-3 regulate the endocytosis of GLR-1 receptors at central synapses (Fig. 9), independent of their role in presynaptic differentiation at NMJs. First, animals with mutations in *rpm-1* accumulate GLR-1 receptors in large accretions, and mutations in *rpm-1* enhance the GLR-1 accumulation observed in *lin-10* mutants. Second, the GLR-1 accretions observed in these mutants colocalize with the endosomal marker Syntaxin-13. Third, while mutations in *rpm-1* result in defective presynaptic boutons at motorneuron NMJs, these same mutations do not appear to alter presynaptic boutons at interneuron central synapses. Fourth, mutations in PMK-3, a p38 MAPK, suppress the accumulation of GLR-1 in endosomal accretions in both *lin-10* and *rpm-1* mutants. Moreover, mutations in PMK-3 suppress the behavioral defects of *lin-10* mutants. Fifth, elevated levels of DLK-1, a MAPKKK upstream of PMK-3, result in a GLR-1 accumulation phenotype similar to that of *rpm-1* mutants. Moreover, DLK-1 levels are negatively regulated by RPM-1 in interneurons. Sixth, mutations resulting in decreased GLR-1 endocytosis occlude the effect of *pmk-3* mutations, whereas transgenic manipulations resulting in increased GLR-1 endocytosis bypass the requirement for PMK-3 and occlude the effect of mutations in *rpm-1* (both on GLR-1 accumulation and GLR-1-mediated behaviors). Based on these results, we propose that RPM-1 regulates GLR-1 trafficking by promoting the ubiquitin-mediated degradation of DLK-1, thereby maintaining reduced levels of PMK-3/p38 MAPK signaling and hence GLR-1 endocytosis.

Mutations in *rpm-1* were originally identified in screens for mutants with aberrant NMJ presynaptic boutons [5], [6]. RPM-1 is expressed in the central as well as the peripheral nervous system, as are its mammalian orthologs Pam and Phr1, but their function in the CNS is unknown. Surprisingly, we found that RPM-1 is not required to organize presynaptic boutons at central synapses; thus, instead of a presynaptic role at central synapses, RPM-1 regulates the endocytosis of AMPARs from postsynaptic elements. This is the first postsynaptic function to be identified for a PHR protein.

While we did not observe obvious presynaptic defects at the central synapses of *rpm-1* mutants, we cannot rule out the possibility that there might be subtle presynaptic defects. Nevertheless, the GLR-1 defects observed in *rpm-1* mutants cannot simply be explained based on a presynaptic role for *rpm-1*. First, the GLR-1 trafficking defect observed in *rpm-1* mutants can be rescued by cell-autonomous, postsynaptic (but not presynaptic) expression of an *rpm-1* minigene. Second, mutants for *unc-104* (KIF1A), which lack most synaptic vesicles from central synapse terminals, do not have the gross GLR-1 localization defects observed in *rpm-1* mutants. In addition, mutants for *unc-18* (Munc18/Sec1), which are deficient in synaptic vesicle release, also are not similar to *rpm-1* mutants [31], [50].

How does RPM-1 function presynaptically in one neuron type and postsynaptically in another? Interestingly, p38 MAPK signaling is the target of RPM-1 regulation at both central synapses and NMJs, suggesting that these genes function as a signaling cassette. One difference for RPM-1 function is the requirement for the GEF GLO-4 and the small GTPase GLO-1 [16]. In motorneurons, RPM-1 acts as an E3 ubiquitin ligase via FSN-1, and as an activator of the GLO-1 Rab via the GLO-4 GEF. Mutations in either *fsn-1* or *glo-4* alone yield mild presynaptic defects, whereas mutations in both are similar to mutations in *rpm-1*, suggesting that RPM-1 relies on both of these factors in parallel to organize NMJ presynaptic boutons. By contrast, mutations in *fsn-1* alone yield the same postsynaptic defects with regard to the trafficking of GLR-1 as do *rpm-1* mutations. Mutations in *glo-1* and *glo-4* appear to have no major postsynaptic defects, suggesting that, in interneurons, RPM-1 acts primarily as an E3 ubiquitin ligase to conduct its postsynaptic function. Thus, the nature of the effecter molecules that associate with RPM-1 in each neuron type might dictate the role of RPM-1 in that neuron.

The p38 MAPK proteins phosphorylate both nuclear and cytosolic targets. In mammals, p38 MAPK regulates endocytosis by phosphorylating components of the endocytosis machinery [43]–[45]. The net effect is increased endocytosis, although how such increased endocytosis is applied to specific cargo is unclear. Similarly, our findings suggest that PMK-3 stimulates GLR-1 endocytosis, perhaps via the activation of RAB-5. It should be noted that we do not have a more direct measure of surface GLR-1, and thus cannot directly address the level of surface GLR-1 in *pmk-3* and *rpm-1* mutants. Indeed, measuring surface receptors directly in a living, intact organism remains an elusive goal for most systems. Nevertheless, we have assayed GLR-1 endocytosis (1) by measuring endogenous GLR-1 via its behavioral function, and (2) by measuring its colocalization with the endosomal marker Syntaxin-13. The findings from these independent approaches reinforce one another, supporting our argument that GLR-1 intracellular trafficking is regulated by *pmk-3* and *rpm-1*.

It should also be noted that, while p38 MAPKs have been shown to regulate endocytosis in the cytosol [43]–[45], we currently cannot rule out a role for PMK-3 in the nucleus of these interneurons. One way to test for a nuclear function of PMK-3 would be to introduce a PMK-3 protein mutated at its nuclear localization signal, then test for the ability of this “cytoplasmically-restricted” PMK-3 mutant protein to rescue GLR-1 trafficking. Unfortunately, the mechanism of p38 MAPK nuclear localization is unknown (p38 MAPKs, including PMK-3, lack a consensus nuclear localization signal), precluding us from conducting such a test [51].

Why does the p38 MAPK cascade require negative regulation by RPM-1? One possibility is that p38 MAPK signaling has to be continually kept in check, with RPM-1 constitutively maintaining low levels of DLK-1 protein. In support of this idea, we found that the levels of DLK-1, but not PMK-3, are limiting with respect to GLR-1 trafficking, suggesting that the activity of this p38 MAPK pathway requires regulation at the MAPKKK step. An alternatively role for RPM-1 might be to act as a feedback to shut off p38 MAPK signaling after the initial activation event. A connection between p38 MAPK activity and RPM-1 levels or ubiquitin-ligase activity would support this model. Finally, in a de-repression mechanism, external stimuli might activate p38 by deactivating RPM-1. To address these questions, we will need a better understanding of both the extrinsic and intrinsic factors that signal via this pathway.

The activation of the PMK-3 pathway by specific extrinsic cues could explain why mutations in *pmk-3* and *rpm-1* in an otherwise wild-type animal yield milder effects on GLR-1 trafficking or GLR-1-mediated behaviors than those observed in *lin-10* mutants: PMK-3 signaling might only be required under certain circumstances. Indeed, an environment-dependent requirement for p38 MAPK in the brain has been observed in knockout mouse strains reared under different environmental conditions [52]. Alternatively, PMK-3 might function redundantly with other pathways; indeed, there are two other p38 MAPK paralogs in the genome [53].

The p38 pathway clearly mediates extrinsic signals in the mammalian brain, including ones that induce both metabotropic glutamate receptor (mGluR)-dependent and NMDAR-dependent long-term depression [44], [54], [55]. In both cases, p38 MAPK is activated by Rap1, which is activated by either G_βγ_ (triggered by mGluRs) or calcium (from NR2B-containing NMDARs) [44], [56]. The p38 MAPK pathway is also activated by reactive oxygen species (ROS) and helps mediate the oxidative stress response in many different tissues [57]. Activated AMPARs can contribute to excitotoxic neuronal death by excessive calcium influx and ROS production [58], [59]. Thus, in addition to the role of p38 MAPK in LTD, it is possible that p38 MAPK is activated by excess ROS, and in turn triggers AMPAR endocytosis. Such negative feedback could protect neurons from excitotoxicity by minimizing oxidative stress. An exploration of AMPAR trafficking in *C. elegans* under conditions of stress or in different environments should help to fully elucidate p38 MAPK signaling in neurons.

## Materials and Methods

Animals were grown at 20°C on standard NGM plates seeded with OP50 *E. coli*. Some strains were provided by the *Caenorhabditis* Genetics Center. All strains were backcrossed multiple times to our laboratory N2 strain to minimize other genetic variation. The following strains were used: N2, *lin-10(e1439)*, *glr-1(ky176)*, *unc-11(e47)*, *rpm-1(ok364)*, *rpm-1(ju41)*, *rpm-1(js317)*, *pmk-3(ok169)*, *dlk-1(ju476)*, *mkk-4(ju91)*, *fsn-1(hp1)*, *odIs22[P_glr-1_::LIN-10::GFP]*, *odIs1[P_glr-1_::SNB-1::GFP]*, *juIs1[P_unc-25_::SNB-1::GFP]*, *nuIs89[P_glr-1_::MUb]*, *nuIs25[GLR-1::GFP],* and the CB4856 Hawaiian strain.

### Isolation and mapping of *lin-10* enhancers

P0 *lin-10 nuIs25* nematodes were EMS mutagenized using standard procedures. F2 animals from individual plates were sampled (n = 30–50) by mounting on 2% agarose pads containing levamisole. Animals were scored by fluorescence microscopy for defects in GLR-1::GFP localization. Mutants were recovered either directly from the slide or by isolating siblings from the parental F1 plate. Mutants were further characterized after 4 rounds of backcrossing.

The *od14* mutation was mapped between *dpy-11* and *vab-8* on the right arm of LGV. A *dpy-11 od14 vab-8* recombinant chromosome was constructed and placed in trans over the CB4856 LGV chromosome to facilitate three-factor SNP mapping. Multiple recombinants placed *od14* between SNP pkP5063 (map position +1.68) and SNP pkP5116 (map position +1.51). This region contains *rpm-1*, and *od14* failed to complement *rpm-1* mutations. A second enhancer, *od22*, was linked to and failed to complement *od14*. We sequenced *rpm-1* genomic DNA from *od14* and *od22* mutants. For *od14*, we identified a missense mutation resulting in a glycine to glutamate change at amino acid 1092, a conserved residue within a PHR domain. For *od22*, we identified a premature stop codon mutation at nucleotide 88 of the predicted *rpm-1* cDNA sequence.

### Transgenes and Germline Transformation

Transgenic strains were isolated after microinjecting various plasmids (5–50 ng/µl) using *rol-6dm* (a gift from C. Mello, UMass) or RFP (monomeric RFP; a gift from R. Tsien, Stanford Univ.) as a marker [60]. Plasmids containing the *glr-1* promoter, followed either by an *rpm-1* minigene, *pmk-3* cDNA, *dlk-1* cDNA, *snn-1*, *syntaxin-13* cDNA, or *rab-5* cDNA (including mutant versions), were generated using standard techniques. The *rab-5(GTP)* and *rab-5(GDP)* cDNAs (mutations Q78L and S23N, respectively) were gifts from B. Grant (Rutgers Univ.). To follow the subcellular localization of PMK-3, SNN-1, Syntaxin-13, and DLK-1 proteins, GFP or RFP sequences were introduced at the N-terminus of the relevant plasmids above. All resulting transgenes were introduced into the germline and followed as extrachromosomal arrays.

### Fluorescence Microscopy

GFP- and RFP-tagged fluorescent proteins were visualized in nematodes by mounting larvae on 2% agarose pads with 10 mM levamisole. Fluorescent images were observed using a Zeiss Axioplan II. A 100× (N.A. = 1.4) PlanApo objective was used to detect GFP and RFP signal. Imaging was done with an ORCA charge-coupled device (CCD) camera (Hamamatsu, Bridgewater, NJ) using IPLab software (Scanalytics, Inc, Fairfax, VA). Exposure times were chosen to fill the 12-bit dynamic range without saturation. Maximum intensity projections of z-series stacks were obtained and out-of-focus light was removed with a constrained iterative deconvolution algorithm. We used a macro written for IPLabs to automatically calculate the outlines of fluorescent objects in ventral cord neurites when they were two standard deviations above the unlocalized baseline fluorescence. This algorithm allowed both the small GLR-1::GFP puncta in wild-type animals and the large, aberrant accretions in *lin-10* and *rpm-1* mutants to be defined as single objects. Object size was measured as the maximum diameter for each outlined cluster. Object number was calculated by counting the average number of clusters per 10 microns of dendrite length.

For the quantification of GFP::DLK-1, mean intensities of transgenic animals were measured from images of cell bodies (captured by a 20× objective) and ventral cords (captured by a 63× objective) using IPLabs software. For both, background signals were subtracted.

For the quantification of GLR-1::GFP and RFP::Syntaxin-13, animals were immobilized on ice for 10 minutes, then fixed with ice cold 1% paraformaldehyde in PBS for 10 min. Images for neuronal cell bodies were taken using a Carl Zeiss confocal microscope equipped with the BD CARV II™ Confocal Imager and a Carl Zeiss 100× Plan-Apochroma objective (N.A. = 1.4). For quantitative colocalization analysis, all image manipulations were performed with iVision v4.0.11 (Biovision Technologies, Exton, PA) software using the FCV colocalization function. We applied an empirically derived threshold to all images for both the GLR-1::GFP channel and the RFP::Syntaxin-13 channel to eliminate background coverslip fluorescence and other noise (typically ∼5% of pixels for each channel). The fluorescent intensity values for both the GLR-1::GFP and RFP::Syntaxin-13 channels were then scatter plotted for each pixel. Pixels with similar intensity values for both channels (within an empirically-established tolerance factor) were counted as colocalized. To acquire the fraction of GLR-1::GFP colocalized with RFP::Syntaxin-13, the number of colocalized pixels was normalized to the number of GLR-1::GFP pixels under threshold. To maximize our resolving power while observing the relatively small *C. elegans* neuron cell bodies, we restricted our analysis to a single focal plane taken through the middle of the cell body.

### Behavioral Assays

The reversal frequency of individual animals was assayed as previously described, but with some modifications [30]. Single young adult hermaphrodites were placed on NGM plates in the absence of food. The animals were allowed to adjust to the plates for 5 minutes, and the number of spontaneous reversals for each animal was counted over a 5-minute period. Twenty animals were tested for each genotype, and the reported scores reflect the mean number of reversals per minute.
